# Elevated serum β2-microglobulin in individuals coinfected with hepatitis B and hepatitis D virus in a rural settings in Southwest Nigeria

**DOI:** 10.1186/s13104-017-3015-9

**Published:** 2017-12-08

**Authors:** Lawrence Ehis Okoror, Ayodele Oluwaseun Ajayi, Oluwaseun Benjamin Ijalana

**Affiliations:** 1 Department of Microbiology, Federal University, PMB 373, Oye-Ekiti, Ekiti State Nigeria; 2 Biosolution Technologies, Olusegun Obasanjo Way, Akure, Nigeria

**Keywords:** 2βmicroglobulin, Hepatitis B, Hepatitis D, ELISA, Coinfection, Monoinfection

## Abstract

**Objective:**

Coinfection of hepatitis B virus (HBV) with hepatitis D virus (HDV) has being reported to increase severity of progression to hepatocellular carcinoma (HCC) and liver cirrhosis (LC). Beta microglobulin (2βM) which is present on the surfaces of blood cells in acceptable levels is a tumor marker which may become elevated in disease conditions. This study hence observed the prevalence of HBV and HDV coinfection in a rural population and their 2βM concentration.

**Results:**

Of the 368 samples, 66 (17.9%) were positive to hepatitis B surface antigen (HBsAg) and 33 (50%) were coinfected with HDV, 8 (2.1%) were monoinfected with HDV. 2βM concentration increased beyond the normal level in individuals coinfected with HBV and HDV as compared with the monoinfected individuals. Coinfection resulted in the increased concentration of 2βM in HBV and HDV coinfection and the likelihood of progression to HCC and LC may not be ruled out. Monoinfection with HDV also had high 2βM concentration but this is due to having being infected with a non-detected HBV or chronic infection in which HBV is clearing.

## Introduction

Hepatitis B virus (HBV) is known to infect approximately 350 million people [1] who are chronic carriers. Over 2 billion people are infected [1, 2] which makes the virus a major public health problem with over 1 million death worldwide. HBV causes diseases of varying severity from acute hepatitis [2] to chronic liver diseases, cirrhosis, hepatocellular carcinoma to fulminant hepatitis [2–5].

HBV is a DNA virus in the family Hepadna viridae, it is highly transmissible so that even a very small quantity of blood is enough to transmit the virus. Major routes of entry of the virus include wounds, abrasions, mucous membranes, bites and scratching [6]. The virus replicates quickly and more rapidly with very high titre in the blood making it more infectious than HIV and hepatitis C, because of this any contact of the blood with the mucosal membranes has a high risk of contracting the virus. The high titre of the virus in the blood makes it easy for transmission via sharp objects like needle stick injuries with a risk of 1–6% if the source of infection is positive for HBV and negative for hepatitis e antigen and 22–40% if positive for both [7]. Risk of mother to child has also been reported [8]. In Nigeria, HBV is endemic with over 12% [9, 10] seropositivity, though Nigerians are said to be chronic carriers, reports vary from population to population. Umolu et al. [11] reported a 5.8% rate in blood donors in Benin City. In a study among university students, Uneke et al. [12] reported 9% seropositivity.

Hepatitis D virus (HDV) is a defective RNA virus which occurs only in HBsAg positive individuals [13–15] as it could only multiply in the presence of HBV infected cells. It has being reported to account for more severe complications in HBV infected individuals leading to rapid progression to cirrhosis, hepatocellular carcinoma and death, unlike those infected with HBV alone [13, 14, 16]. The occurrence of HDV could be as a result of super infection of chronic HBV infection or they could co-occur in the acute infection [16].

In Nigeria information regarding HDV is scanty but a few have reported HDV in chronic liver diseases [17]. Due to the complications likely to be produced by HBV and HDV confection, more awareness needs to be created in Nigeria and sub-Saharan Africa.

Beta-2-microglobulin is one of the major histocompatibility complex class molecules on the cell surfaces of all nucleated cells. It interacts and stabilizes the tertiary structure of the major histocompatibility complex class 1 alpha-chain [18]. It is a member of the human leucocyte antigen (HLA) where it is expressed in certain diseases conditions such as tumor in elevated concentration in the blood and detected in serum. Hepatitis D virus infection in HBV infected individuals increases the chances of development to hepatocellular carcinoma which may be seen in the elevated level or concentration of 2βM.

We report the co-occurrence of HBV and HDV in relation to their 2βM concentration in a rural population. To our knowledge this is the first study in Nigeria to use 2-β-microglobulin to evaluate the state of HBV/HDV co-occurrence.

## Main text

### Materials and methods

A total of 368 blood samples were collected from apparently healthy individuals in a rural suburb in South West Nigeria. Participants included in this study were either HBV positive or HDV positive or both and must have lived in the study population for the past 1 year. All participant confirmed participation, parental consent was obtained for children below the age of 18. HBV, HDV mono-infected individuals served as controls.

### Sample processing

Sera were separated from whole blood by centrifuging at 3000 rpm and tested for hepatitis B surface antigen (HBsAg), HDV and 2βM using the quantitative ELISA technique for all the 3 parameters and test performed as directed by the manufacturer of the ELISA kit (WKEA Medical Supplies, China). Absorbance of all the samples was read on a microplate reader (Thermomax, Molecular Devices, USA) at 450 nm optical density. HBsAg, HDV and 2βM concentration was determined using curves from myassays ELISA analysis on line software for all samples plotted against the standards.

### Statistical analysis

Data analysis was done using the openepi which is an online epidemiological software and SAS University Edition determining seroprevalence of HDV in HBsAg positive patients and expressed as percentage of the study group/Pearson Chi square was used to determine the association between the concentration of 2βM and HBV/HDV infection. Linear correlation analysis was used to determine the association between age and the concentration of 2βM and HBV/HDV infection.

### Results

**Fig. 1:**
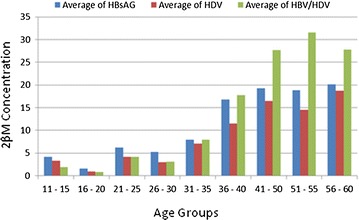
Average concentration of 2βM along the age groups of patients positive to HBsAg and HDV coinfection and HBsAg and HDV monoinfection

**Fig. 2:**
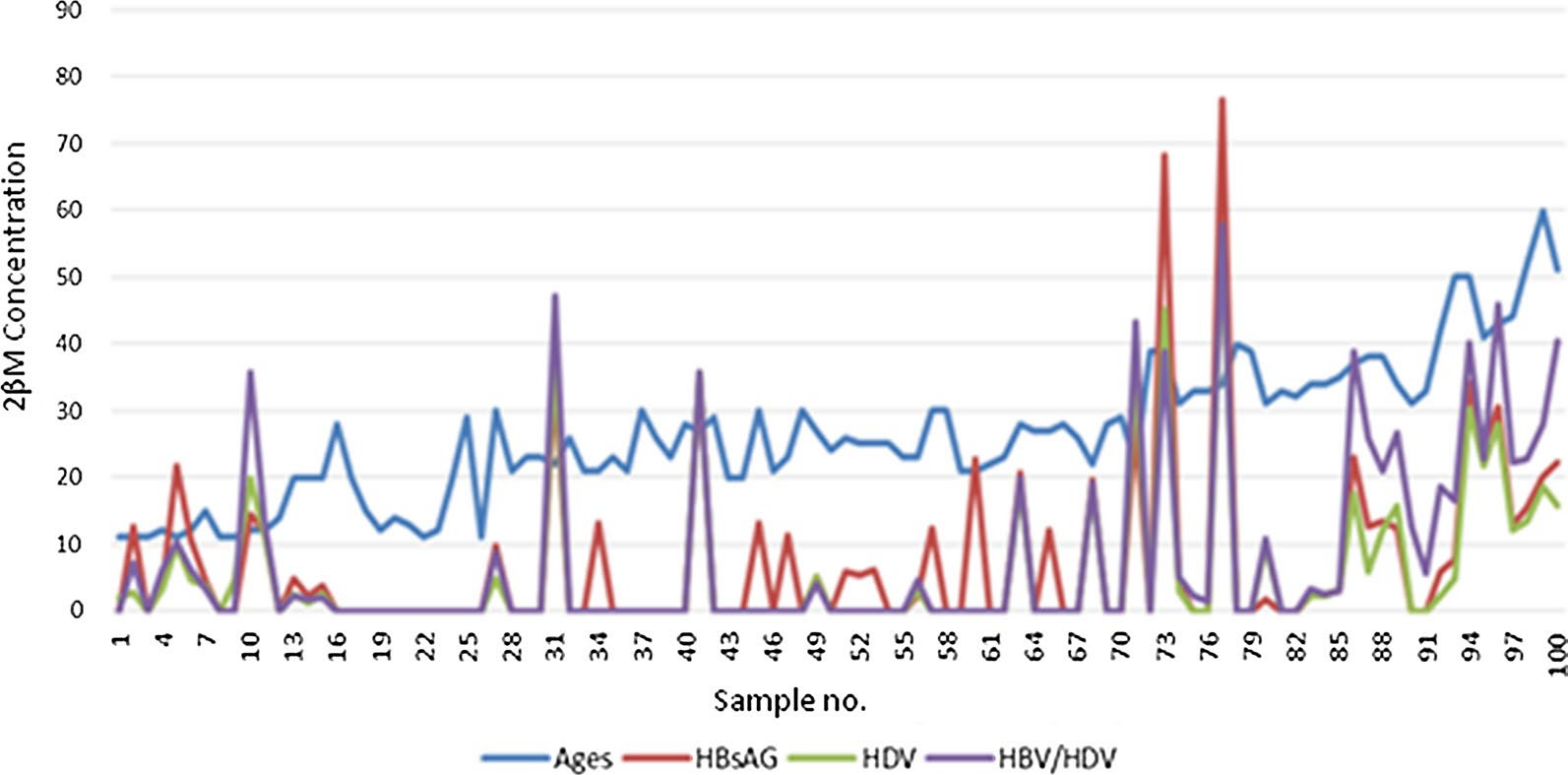
Concentration of 2βM in individuals positive to HBV, HDV and HBV/HDV coinfection and as related to their individual ages

Of the 368 samples tested for HBsAg, 240 (65.2%) were females while 128 (34.8%) were males, 66 (17.9%) were positive to HBsAg of which 39 (59.1%) were females and 27 (40.9%) were males. A total of 33 (50%) individuals were coinfected with HBV and HDV of which 8 (24.2%) were males while 25 (75.8) were females. Eight individuals (2.1%) were monoinfected with HDV from which there were 4 (50%) males and 4 (50%) females (Table 1). Age group 21–30 had the highest number of individuals 29 (12.9%), they also have the highest number of individuals testing positive to HBV/HDV coinfection. Chi square (χ^2^ = 271.9; p ≤ 0.0000001) shows that there is significant difference between all results across the table. Figure 2 confirms the increasing level of 2βM with HBV/HDV coinfection. The average number of individuals coinfected with HBV and HDV with elevated 2βM was more in age groups 56–60, 51–55 and 41–50 (Fig. 1) (χ^2^ = 271.9; p ≤ 0.0000001).

**Table 1:** Age distribution of HBV, HDV monoinfected and HBV/HDV coinfected individuals with significant 2βM concentration

Characteristics	Total tested		HBsAg (+ve)		HDV (+ve)		HBsAg/HDV (+ve)		Total (+ve)	Mean	CI
Age groups	Male	Female	Male	Female	Male	Female	Male	Female			
11–20	8	56	9	9	0	2	0	5	25	25.33	42.13
21–30	80	144	8	21	1	1	6	10	47	78.67	126.55
31–40	16	24	1	9	1	0	1	9	20	26.44	39.80
41–50	16	8	3	0	1	0	1	1	6	12	18.87
51–60	8	8	1	0	1	1	0	0	3	6	9.57
Total	128	240	27	39	4	4	8	25	101	148.44	231.87

χ^2^ = 271.9; p = < 0.0000001

### Discussion

The major finding in this study is the significant increase in 2βM concentration in HBV/HDV coinfection (Fig. 1) compared with HBsAg monoinfection and HDV monoinfection. Yegane et al. [17] reported the role of 2βM in monitoring chronic HBV infection where they estimated the serum 2βM level in individuals with chronic HBV infection and found that serum 2βM was higher in cases of chronic HBV as compared to controls without HBV. Though they did not work on HBV/HDV coinfection, their observation was similar to the result obtained in this study as 23.7% of patients monoinfected with HBV, 29% monoinfected with HDV and 39% coinfection have 2βM level well above the acceptable serum concentration and high incidence of HDV in chronic HBV has being reported. Yeagane et al. [17] then concluded that 2βM is an indicator for monitoring chronic hepatitis which is in agreement with this study as the trend of increase suggests that coinfection of HBV/HDV have influenced the increment in 2βM concentration in coinfected individuals. This result is similar to a study by Casey et al. [18] where they reported 64% of individuals were coinfected with HBV and HDV and concluded that coinfection of HBV with HDV was responsible for the frequent hepatitis outbreak in a military setting. Wanch et al. [19] reported the influence of age in elevated 2βM concentration in patients infected with tuberculosis with the older ages having higher concentration. Garcia-Garcia et al. [20] also confirmed this report which was also seen in this study as the older age groups had higher concentration of 2βM which may be due to longer exposure to HBV and becoming chronic as against the younger population who has just recently been exposed. Figure 2 shows individual 2βM concentration from the 101 positive individuals for all the 3 parameters tested and age, individuals from the older age groups had higher concentration of 2βM though prevalence was higher in mid ages had more individuals showing elevated 2βM levels. There was more elevated concentration of 2βM in individuals with coinfection of HBV and HDV than those with monoinfection in the mid ages. Though there were cases in this study with high 2βM concentration in monoinfected individuals, this was actually noticed in people with older ages and could have been due to other cofounders as earlier reported by Wanch et al. [19] and chronic HBV infection. Figure 2 also reveals a positive correlation between age and HBV and HDV monoinfection (r = 0.8912; CI 95%). This also confirmed that as age increases 2βM concentration increases in individuals coinfected with HBV/HDV in significant proportion. This is also supported by reports from Wanch et al. [19] where they reported that age has an influence in the increase in 2βM concentration in patients infected with tuberculosis with the older ages having more prevalence. Garcia-Garcia et al. [20] also confirmed this report. This study further reports that HBV is still a major public health concern which case is worsened by HDV infection especially in the rural areas with limited health facilities and public health awareness. Findings in this study when compared to earlier reports shows that HBV is actually on the rise especially in the rural areas. Umolu et al. [11] reported 5.8% in blood donors while Uneke et al. [9] reported 9% seropositivity among university students. These two reports dealt with enlightened population where there are health facilities and public health awareness. The chances of progression to HCC and LC is more in the rural areas as evident in this results from this study with the involvement of HDV and the elevated 2βM. Though this is the first report using 2βM to monitor HBV/HDV coinfection in Nigeria.

This study also reveals a rise in HBV infection (17.6%) despite available vaccines against the virus in Nigeria a rise from Umolu et al. [15] and Alao et al. [12] who separately reported over 12% HBsAg which was similar to what was obtained in this study; however the trend is increasing. Udeze et al. [21] reported a 9.0% HBsAg positivity among university students in Nigeria a report which is lower than the 17.9% from this study this can be said to be due to different population studied. An Egyptian study reported 21% prevalence [11]. This rise in prevalence in HBV infection despite the vaccination campaign may also be as a result of increasing population and also the likely involvement of other immune suppressing diseases not captured in this study. There may be a possibility of reduction of HBV in the population in the near future due to the vaccination campaign but there could be high prevalence of HBV-associated LC and HCC due to long latency of chronic hepatitis which eventually involve the HDV coinfection which was earlier reported to be due to chronic HBV infection leading to rapid progression to HCC and LC.

Eight individuals (2.1%) were monoinfected with HDV from which there were 4 (50%) males and 4 (50%) females (Table 1) this we suggest have resulted from HBV chronic infection. Though there is paucity of reports on monoinfection with HDV, a few reports have it that monoinfection with HDV may occur in individuals who had HBV infection earlier and may lead to serious sequelae where it becomes persistence and develops to hepatocellular carcinoma more severe and quicker than a coinfection with HBV since it may have resulted as a result of chronic HBsAg. Katja et al. [22] inoculated mice with HDV and concluded that HDV could persist even in humans for over 8 weeks before being rescued by HBV and thereby develop more virulence. However, mechanism for HDV monoinfection is still being investigated.

### Conclusion

This study confirms elevated 2βM concentration in HBV/HDV coinfected individuals. It is also suggested that 2βM concentration could also be used as a marker to complications that could arise due to HBV/HDV coinfection. We also conclude that there is little awareness campaign in the rural areas as to danger posed by HBV and or HBV/HDV coinfection. Therefore awareness campaign in the rural communities must be stepped up in order to reduce the rising HBV/HDV infection.

## Limitations

- We were unable to assay for other debilitating diseases that could lead to immunocompromise and then enhance HBV infection.
- We did not confirm other diseases that could cause increase in 2βM concentration but this did not undermine our results as we have HBV and HDV monoinfection served as controls.
- Confounding factors like chemicals not covered in this study are likely to cause elevated 2βM though there influence were taking care of by controls.

## Abbreviations

2βM: 2-beta microglobulin
HBV: hepatitis B virus
HDV: hepatitis D virus
HCC: hepato cellular carcinoma
LC: liver cirrhosis
HBsAg: hepatitis B surface antigen
DNA: deoxyribonucleic acid
RNA: ribonucleic acid
HLA: human leucocyte antigen
ELISA: enzyme linked immunosorbent assay
